# Supplementary material for: Health in Chile’s Recent Constitutional Process: A Qualitative Thematic Analysis of Civil Proposals
Source: Int J Environ Res Public Health. 2022 Dec 16;19(24):16903. doi: 10.3390/ijerph192416903 (PMC9778835; doi:10.3390/ijerph192416903)
Supplement: Supplementary file 1 [file ijerph-19-16903-s001.zip › ijerph-1958705-supplementary.pdf]

Supplementary file 1.
Full list of civil proposals included in this study.

Title	Main idea	Institutional support	Number of votes	Health Goals 2030 Strategic lines (Chile)	Sustainable Development Goals
Mental health (13)					
Right to free and quality mental health	Citizens will have the right to free and optimal mental health services.	-	20590	2.Lifestyle<br>4.Non communicable chronic diseases and Violence<br>5. Development and Disability<br>7. Management, quality and innovation	3. Good Health and Well-Being<br><br>10. Reduced Inequalities
A right to mental health, promotion of wellbeing, dignified and inclusive mental health services. May no one be left behind	Mental health must be guaranteed, as an integral and inseparable element of health	"Right to Mental Health Collective"	1755	2.Lifestyle<br>4.Non communicable chronic diseases and Violence<br>5. Development and Disability<br>7. Management, quality and innovation	3. Good Health and Well-Being<br><br>10. Reduced Inequalities
Mental health as a right and not a privilege	Mental health must be considered a guaranteed right, therefore all diseases will be covered by the state, including service, quality, treatment, referrals and instalments.	-	1699	2.Lifestyle<br>4.Non communicable chronic diseases and Violence<br>5. Development and Disability<br>7. Management, quality and innovation	3. Good Health and Well-Being<br><br>10. Reduced Inequalities
Right to mental health with no discrimination, in equal	The constitution must recognize the right to universal, free and quality mental	-	1175	2.Lifestyle	3. Good Health and Well-Being

conditions to physical health	health, without exclusions or stigmas, guaranteeing the protection of human rights and considering social determinants of health, cultural differences and gender.			4.Non communicable chronic diseases and Violence<br>5. Development and Disability<br>7. Management, quality and innovation	5. Gender Equality<br><br>10. Reduced Inequalities
Timely access to free, quality mental health. A patient should never have to say "I have been waiting for an appointment for months"	To ensure the right to mental health for all people, independently of their economic situation	-	827	2.Lifestyle<br>4.Non communicable chronic diseases and Violence<br>5. Development and Disability<br>7. Management, quality and innovation	3. Good Health and Well-Being<br><br>10. Reduced Inequalities
Strengthening mental health	To generate public policy for promotion of mental health in education, health and work areas, as well as equal treatment conditions.	-	774	1. Environmental Health and Healthy Environments<br>2.Lifestyle<br>4.Non communicable chronic diseases and Violence<br>5. Development and Disability<br>7. Management, quality and innovation	3. Good Health and Well-Being<br><br>10. Reduced Inequalities
Mental health as a constitutional norm: Guaranteeing the universal right to protection of mental health	Health must be considered a right, with emphasis on the participation of local communities, sustained on a human rights focus.	Health Mind NGO	644	1. Environmental Health and Healthy Environments<br>2.Lifestyle<br>4.Non communicable chronic diseases and Violence<br>5. Development and Disability<br>7. Management, quality and innovation	3. Good Health and Well-Being<br><br>10. Reduced Inequalities

Right to quality mental health for everyone	The state must guarantee mental health equally to general health, therefore FONASA and ISAPRE must cover psychological and psychiatric services the same way as other specialties.	-	537	2.Lifestyle<br>4.Non communicable chronic diseases and Violence<br>5. Development and Disability<br>7. Management, quality and innovation	3. Good Health and Well-Being<br><br>10. Reduced Inequalities
Free, quality mental health as a fundamental right, and decentralised priority for Chileans	To ensure the right to free mental health, guaranteed by a decentralised, national coverage.	-	323	1. Environmental Health and Healthy Environments<br>2.Lifestyle<br>4.Non communicable chronic diseases and Violence<br>5. Development and Disability<br>7. Management, quality and innovation	3. Good Health and Well-Being<br><br>10. Reduced Inequalities
Right to community mental health: closure of psychiatric hospitals	To guarantee community based health, associated with the territory and communities, ensuring self-determination and cultural pertinence, as well as progressively closing psychiatric hospitals so that these patients may be cared for in a community-based model.	-	378	1. Environmental Health and Healthy Environments<br>2.Lifestyle<br>4.Non communicable chronic diseases and Violence<br>5. Development and Disability<br>7. Management, quality and innovation	3. Good Health and Well-Being<br><br>10. Reduced Inequalities
Inalienable right to prevention, detection, diagnosis, attention, treatment and education in mental health	A state policy must ensure prevention, containment, assistance, adequate treatment and permanent education in relation to mental health	-	298	1. Environmental Health and Healthy Environments<br>2.Lifestyle<br>4.Non communicable chronic diseases and Violence<br>5. Development and Disability	3. Good Health and Well-Being<br><br>10. Reduced Inequalities

				7. Management, quality and innovation	
The state's responsibility of encouraging, promoting and guaranteeing quality mental health attention for all Chileans	The state is in charge of promoting, preventing and guaranteeing mental health attentions that citizens may need	-	293	2.Lifestyle<br>4.Non communicable chronic diseases and Violence<br>5. Development and Disability<br>7. Management, quality and innovation	3. Good Health and Well-Being<br><br>10. Reduced Inequalities
Towards the integration of communitarian mental health	The state must guarantee the right to mental health, increasing coverage and diversifying the focus from an individual to a communitarian approach	-	70	1. Environmental Health and Healthy Environments<br>2.Lifestyle<br>4.Non communicable chronic diseases and Violence<br>5. Development and Disability<br>7. Management, quality and innovation	3. Good Health and Well-Being<br><br>10. Reduced Inequalities
Mental and dental health (2)					
Free physical, mental and dental health	The constitution will include free and quality physical, dental and mental health.	-	418	1. Environmental Health and Healthy Environments<br>2.Lifestyle<br>4.Non communicable chronic diseases and Violence<br>5. Development and Disability<br>7. Management, quality and innovation	3. Good Health and Well-Being<br><br>10. Reduced Inequalities
Guaranteed access to quality and free mental and	Every citizen will have free and equal access to mental and dental health,	-	169	1. Environmental Health and Healthy Environments	3. Good Health and Well-Being

dental health at a community level, for everyone, independently of their socioeconomic level	independently of their age, gender, socioeconomic level or region.			2.Lifestyle<br>4.Non communicable chronic diseases and Violence<br>5. Development and Disability<br>7. Management, quality and innovation	10. Reduced Inequalities
Dental health (6)					
Right to dental health	To incorporate the right to dental health, specifying that health systems must add it into their coverage.	-	2964	4.Non communicable chronic diseases and Violence<br>7. Management, quality and innovation	3. Good Health and Well-Being<br><br>10. Reduced Inequalities
Oral health as a fundamental right for the elderly population	To establish dental health as a fundamental right for our population, including elderly population.	Centre of Epidemiology and and surveillance of Oral Diseases	809	4.Non communicable chronic diseases and Violence<br>7. Management, quality and innovation	3. Good Health and Well-Being<br><br>10. Reduced Inequalities
Oral health as a right	Oral health will be recognized as a fundamental component of people's health and therefore must be incorporated as a right.	-	665	4.Non communicable chronic diseases and Violence<br>7. Management, quality and innovation	3. Good Health and Well-Being<br><br>10. Reduced Inequalities
The right to oral health for all Chileans	The right to oral health must be Universal, Plurinational and Integrated to the community, by establishing right to health and implementing a single health system	-	529	1. Environmental Health and Healthy Environments<br>4.Non communicable chronic diseases and Violence	3. Good Health and Well-Being<br><br>10. Reduced Inequalities

				7. Management, quality and innovation	
Dental health, urgently	To guarantee quality and efficient dental health to anyone who needs it, free of charge or partially covered through FONASA.	-	438	4.Non communicable chronic diseases and Violence<br>7. Management, quality and innovation	3. Good Health and Well-Being<br><br>10. Reduced Inequalities
Dental health guaranteed in case of loss of dental pieces	It must be the States duty to guarantee that people have access to dental health in the case of losing a tooth or dental pieces	-	169	4.Non communicable chronic diseases and Violence<br>7. Management, quality and innovation	3. Good Health and Well-Being<br><br>10. Reduced Inequalities
Public employees (4)					
Public authorities and staff must use public health, education and transport services	Public authorities and staff must use public health, education and transport services	-	2911	1. Environmental Health and Healthy Environments<br>7. Management, quality and innovation	3. Good Health and Well-Being<br><br>10. Reduced Inequalities
State health care for all public workers	Every public employee must obligatorily be affiliated to the National Health Fund (FONASA)	-	1074	1. Environmental Health and Healthy Environments<br>7. Management, quality and innovation	3. Good Health and Well-Being<br><br>10. Reduced Inequalities
Government authorities and staff must use public health and education systems	Government authorities and staff, along with their families, must use the Public Health and Education Systems.	-	611	1. Environmental Health and Healthy Environments<br>7. Management, quality and innovation	3. Good Health and Well-Being

					10. Reduced Inequalities
Quality public health	Every public employee must use the Public Health System	-	295	1. Environmental Health and Healthy Environments<br>7. Management, quality and innovation	3. Good Health and Well-Being<br><br>10. Reduced Inequalities
Equal health for all	All health professionals must attend FONASA patients, without exclusion.		225	1. Environmental Health and Healthy Environments<br>7. Management, quality and innovation	3. Good Health and Well-Being<br><br>10. Reduced Inequalities
Child and adolescent health (5)					
Healthcare within educational institutions	Health education and healthcare assessments must be present within educational institutions	Chilean Society of Nursing in School Health	830	1. Environmental Health and Healthy Environments<br>5. Development and Disability<br>7. Management, quality and innovation	3. Good Health and Well-Being<br><br>4. Quality Education<br><br>10. Reduced Inequalities
Guaranteed mental health for our children and youth	Mental health must be guaranteed, to give a real solution to disorders which lead to juvenile delinquency	-	513	1. Environmental Health and Healthy Environments<br>2. Lifestyle<br>4.Non communicable chronic diseases and Violence<br>5. Development and Disability	3. Good Health and Well-Being<br><br>10. Reduced Inequalities

				7. Management, quality and innovation	
Strength conditioning and functional training for middle school students, to strengthen physical and mental health and gender equality	Athletic and functional training workshops must be implemented for middle school students, incentivizing collaborative work and improving overall health.	Open Community	224	1. Environmental Health and Healthy Environments<br>2. Lifestyle<br>4.Non communicable chronic diseases and Violence	3. Good Health and Well-Being<br><br>4. Quality Education<br><br>5. Gender Equality<br><br>10. Reduced Inequalities
Every child has a right to have a good quality of life and the state must propitiate and guarantee adoption systems and capacitation centres	Children must be guaranteed their fundamental rights. The state must provide safe environments and expedient adoption processes, along with the right to a proper mental health for those in vulnerable situations	-	137	1. Environmental Health and Healthy Environments<br>2. Lifestyle<br>4.Non communicable chronic diseases and Violence<br>7. Management, quality and innovation	3. Good Health and Well-Being<br><br>10. Reduced Inequalities
Equalitarian and independent access to health care for children and adolescents	Access to health care must be guaranteed for this group, without requiring adult authorization, when a minor considers it to be necessary.	-	53	1. Environmental Health and Healthy Environments<br>7. Management, quality and innovation	3. Good Health and Well-Being<br><br>10. Reduced Inequalities
Autonomous organisms for health and education (3)					
Autonomous health and education	There must be efforts to advance in the decentralisation of power and in the	-	91	1. Environmental Health and Healthy Environments	3. Good Health and Well-Being

	modernization of the state with regards to health and education, that must work independently of the government in place.			7. Management, quality and innovation	4. Quality Education<br><br>10. Reduced Inequalities
Autonomy for education and health	Education and health must be considered state policies with long term objectives, independently of the changes in government, lead by a technical, fiscalized committee	-	85	1. Environmental Health and Healthy Environments<br>7. Management, quality and innovation	3. Good Health and Well-Being<br><br>4. Quality Education<br><br>10. Reduced Inequalities
Establishment of autonomous organisms for health and education	State policies, in terms of health and education, must be independent of the executive power, allowing for long term economic and political measures.	-	31	1. Environmental Health and Healthy Environments<br>7. Management, quality and innovation	3. Good Health and Well-Being<br><br>4. Quality Education<br><br>10. Reduced Inequalities
Health, sustainability and environment (5)					
Health and quality of life	Quality of life must be guaranteed by not allowing commercial airplanes to transit through densely populated areas		6005	1. Environmental Health and Healthy Environments	3. Good Health and Well-Being

					10. Reduced Inequalities<br><br>11. Sustainable Cities and Communities<br><br>13. Climate Action
The right to health in an environment free of contamination	The state must norm the presence of contaminants, considering the well being and health of people and ecosystems.	United for Responsible Technology	712	1. Environmental Health and Healthy Environments	3. Good Health and Well-Being<br><br>10. Reduced Inequalities<br><br>11. Sustainable Cities and Communities<br><br>13. Climate Action<br><br>15. Life on Land
Right to a good living through the protection against acoustic contamination	The right to be protected against acoustic contamination must be ensured, considering its adverse effect on health	-	370	1. Environmental Health and Healthy Environments	3. Good Health and Well-Being<br><br>10. Reduced Inequalities

					11. Sustainable Cities and Communities<br><br>15. Life on Land
The right to health in an environment with reduced contamination	The presence of contaminants in technology must be regulated by the state, ensuring the right to life and physical and psychological integrity for its citizens	United for Responsible Technology	268	1. Environmental Health and Healthy Environments	3. Good Health and Well-Being<br><br>10. Reduced Inequalities<br><br>11. Sustainable Cities and Communities<br><br>13. Climate Action<br><br>15. Life on Land
Without a right to food, there is no right to health nor healthy development	The state must advance in guaranteeing the right to proper food and health food environments, ensuring accessibility and sustainability.	Transdisciplinary group for Obesity within populations (GTOP)	108	1. Environmental Health and Healthy Environments<br>2. Lifestyle<br>4.Non communicable chronic diseases and Violence	2. Zero Hunger<br><br>3. Good Health and Well-Being<br><br>10. Reduced Inequalities

Women, sexual and reproductive health rights (8)					
It will be law	The state must regulate and guarantee a free and informed exercise of sexual and reproductive rights, including the right to have an abortion. The guiding principles for these clauses will be Autonomy, Freedom, Dignity and Substantial Equality.	Permanent Assembly for the Legalization of Abortion	38198	2. Lifestyle<br>7. Management, quality and innovation	3. Good Health and Well-Being<br><br>5. Gender Equality<br><br>10. Reduced Inequalities
Always for life	The constitution will recognize the right to life and physical and psychological integrity since conception, and the law will protect the life of unborn beings.<br>Scientific and technological development will be at the disposal of people and will be carried out with repect to life in its physical and psychic aspects.	Always for life	31208	2. Lifestyle<br>7. Management, quality and innovation	3. Good Health and Well-Being
Right to sexual and reproductive health with a gender, feminist, intersectional and pluralist focus	To replace the current legal regulations for a new law that guarantees the right to sexual and reproductive health with a gender, feminist, intersectional and pluralist focus	Asociación Nacional de Matronas y Matrones de Chile AG.	15558	2. Lifestyle<br>7. Management, quality and innovation	3. Good Health and Well-Being<br><br>5. Gender Equality<br><br>10. Reduced Inequalities
Reproductive rights and access to assisted reproduction in the new	People and couples must have the right to access the necessary technology to have	Sociedad Chilena de Medicina Reproductiva	4492	2. Lifestyle<br>7. Management, quality and innovation	3. Good Health and Well-Being

Chile: a proposal from civilians and scientific societies	children, independently of their sexual, social or economic status.				5. Gender Equality<br><br>10. Reduced Inequalities
¡Right to sexual health!	The state must recognize people as sexual beings and respect their sexual rights, implementing: health services for issues in sexual health, training health and education students in this area and providing integral sexual education for the population.	Escuela Transdisciplinaria de Sexualidad	712	2. Lifestyle<br>7. Management, quality and innovation	3. Good Health and Well-Being<br><br>5. Gender Equality<br><br>10. Reduced Inequalities
Guarantee the right to voluntary contraception within the framework of protecting sexual and reproductive rights	To guarantee permanent, free, safe and quality access to contraceptive methods		615	2. Lifestyle<br>7. Management, quality and innovation	3. Good Health and Well-Being<br><br>5. Gender Equality<br><br>10. Reduced Inequalities
Respected gestation and birth	To integrate doulas and other complementary therapies to the current health system. The state would play a role in providing information as well as ensuring that doulas could be present in different stages of gestation, birth, breastfeeding and postpartum.		375	2. Lifestyle<br>7. Management, quality and innovation	3. Good Health and Well-Being<br><br>5. Gender Equality<br><br>10. Reduced Inequalities
Access to permanent menstrual education and	To advance towards a state that promotes, protects and guarantees the menstrual	World Supports Women (MAM)	217	2. Lifestyle	3. Good Health and Well-Being

health for girls, women and menstruating people in Chile	rights of girls, women and menstruating people.			7. Management, quality and innovation	5. Gender Equality<br><br>10. Reduced Inequalities
Conscientious objection to abortion for healthcare workers	Conscientious objection to abortion for healthcare workers will be allowed for those who, for ethical or personal reasons, do not wish to be part of abortion related procedures.		351	2. Lifestyle<br>7. Management, quality and innovation	
Palliative, chronic pain and end of life care (4)					
"Care and health protection in the new constitution"	To establish the right to receive and the duty to give care, as a fundamental part of health promotion, prevention and maintenance.	National Federation of Nurses of Chile	1332	1. Environmental Health and Healthy Environments<br>2. Lifestyle<br>4.Non communicable chronic diseases and Violence<br>7. Management, quality and innovation	3. Good Health and Well-Being<br><br>10. Reduced Inequalities
Right to "good death"	There will be trained palliative care teams financed by the state, capacitated to help gravely ill people, with access to medication and drugs that allow a dignified and opportune death.		496	1. Health and Healthy Environments<br>5. Development and Disability<br>7. Management, quality and innovation	3. Good Health and Well-Being<br><br>10. Reduced Inequalities
Mental health and wellbeing of elderly patients and their carers.	Caring for the elderly must be a professional, remunerated activity and every elderly person in the country will be		269	1. Environmental Health and Healthy Environments<br>5. Development and Disability	3. Good Health and Well-Being

Professionalising the care of the elderly	able to access proper medical attention in their last years of life.			7. Management, quality and innovation	10. Reduced Inequalities
"Right to live without pain": for a compassionate treatment of chronic, non-oncological pain	The right to live without pain will be recognized through ensuring the effective right of people to have integral physical and psychological health.		226	1. Environmental Health and Healthy Environments<br>5. Development and Disability<br>7. Management, quality and innovation	3. Good Health and Well-Being<br><br>10. Reduced Inequalities
Medication, specific treatments and alternative therapies					
Medication (3)					
Access to quality medication and pharmacies as health centres	Medications, vaccines and medical supplies must be considered as essential goods, and therefore access to them must be guaranteed. Additionally, pharmacies must be considered part of the healthcare network	Colegio de Químicos Farmacéuticos y Bioquímicos de Chile A.G.	6026	1. Environmental Health and Healthy Environments<br>3. Communicable Diseases<br>4.Non communicable chronic diseases and Violence<br>7. Management, quality and innovation	3. Good Health and Well-Being<br><br>10. Reduced Inequalities
Universal and free access to all remedies and treatments, independently of the disease	To establish and guarantee a system of universal and free access to all remedies and medications, independently of the cost or the disease, financed by the state.	-	1210	1. Environmental Health and Healthy Environments<br>3. Communicable Diseases<br>4.Non communicable chronic diseases and Violence<br>7. Management, quality and innovation	3. Good Health and Well-Being<br><br>10. Reduced Inequalities
Medication as an essential article of public use, and	Medication will be considered an essential good of public use, and pharmacies will be used as primary care health centres	-	330	1. Environmental Health and Healthy Environments<br>3. Communicable Diseases	3. Good Health and Well-Being

pharmacies as primary health centres				4.Non communicable chronic diseases and Violence<br>7. Management, quality and innovation	10. Reduced Inequalities
Specific treatments (6)					
Obesity and loose skin: our health is your responsibility	The state must promote healthy lifestyles but also be in charge of solving problems such as "guatita de delantal" (loose abdominal skin) post surgeries such as abdominoplasties.	Guatita Delantal Movement Association	12936	1. Environmental Health and Healthy Environments<br>4.Non communicable chronic diseases and Violence<br>7. Management, quality and innovation	3. Good Health and Well-Being<br>10. Reduced Inequalities
Right to physical therapy and rehabilitation to face disability - School of Physiotherapists	The state must guarantee opportune and quality access to physical therapy and rehabilitation to all citizens in national territory	School of Physiotherapists of Chile	8225	1. Environmental Health and Healthy Environments<br>4.Non communicable chronic diseases and Violence<br>5. Development and Disability<br>7. Management, quality and innovation	3. Good Health and Well-Being<br>10. Reduced Inequalities
Organ donation and transplants and their promotion as a public policy	It will be the states responsibility to guarantee access to information and complete treatment for whoever needs an organ transplant, therefore it must establish means to ensure sufficient resources	Let's Change History Foundation	1234	4.Non communicable chronic diseases and Violence<br>7. Management, quality and innovation	3. Good Health and Well-Being<br>10. Reduced Inequalities
Blood as a common good and its equal and opportune	To incorporate altruistic blood donation as a constitutional guarantee, ensuring	Community of Altruist Donors of	706	4.Non communicable chronic diseases and Violence<br>5. Development and Disability	3. Good Health and Well-Being

availability for all inhabitants of the country	opportune, efficient and equal blood transfusions.	the Southern Network		7. Management, quality and innovation	10. Reduced Inequalities
Creation of a Nation Organ Transplant Institution	Crear y garantizar la autonomía de una institución que vele por la coordinación y trasparencia de los trasplantes de órganos en Chile		154	4.Non communicable chronic diseases and Violence<br>5. Development and Disability<br>7. Management, quality and innovation	3. Good Health and Well-Being<br>10. Reduced Inequalities
Right to quality esthetic health for everyone	The state must regulate and finance health services destined to improve the population's esthetic health.		92	4.Non communicable chronic diseases and Violence<br>5. Development and Disability<br>7. Management, quality and innovation	3. Good Health and Well-Being<br>10. Reduced Inequalities
Alternative/Complementary therapies (7)					
Cannabis to the Constitution now: For the right to freedom in the development of personality, personal sovereignty and wellbeing	Every person will have the right to cultivate a bond with nature in a balanced and respectful way. In this line, the state will respect people's personal freedom, autonomy, their privacy and search for wellbeing, pleasure and integral health, including the choice to use cannabis and other psychoactive substances of natural or synthetic origins.	Cannabis Activism Chile	44332	1. Environmental Health and Healthy Environments<br>2. Lifestyle	3. Good Health and Well-Being<br>10. Reduced Inequalities<br>11. Sustainable Cities and Communities<br>13. Climate Action

					15. Life on Land
Acupuncture - Chinese Medicine - Integrative therapies	Chinese Medicine and Acupuncture must be recognized as part of the medical services within the Sanitary Code, giving people the freedom to choose this alternative, with complete coverage from FONASA or the alternative insurance.	Professional School of Acupuncturists Chile A.G.	4140	1. Environmental Health and Healthy Environments<br>2. Lifestyle<br>7. Management, quality and innovation	3. Good Health and Well-Being<br><br>10. Reduced Inequalities
Floral therapy as a constitutional right	Floral therapy must be a real option of treatment, with free and timely access.	National Association of Floral and Natural Therapists, Radiata AG	1603	1. Environmental Health and Healthy Environments<br>2. Lifestyle<br>7. Management, quality and innovation	3. Good Health and Well-Being<br><br>10. Reduced Inequalities
Health and natural medicine: a necessity for Chile	The constitution must state that every person will have the right to choose the health system they wish to benefit from, be it state or private, recognizing natural medicine as an option.	Non-profit Association: "Natural Project"	1493	1. Environmental Health and Healthy Environments<br>2. Lifestyle<br>7. Management, quality and innovation	3. Good Health and Well-Being<br><br>10. Reduced Inequalities
Health centred on the human being, with integrative and complementary medicine	The State recognizes and incorporates traditional medicine, complementary medicine and health welfare practices within the universal health system,		802	1. Environmental Health and Healthy Environments<br>2. Lifestyle<br>7. Management, quality and innovation	3. Good Health and Well-Being

	including them as part of the Fundamental Right to Health.				10. Reduced Inequalities
Right to access health care through conventional, traditional or complementary medicine, according to choice	People must have the right to choose health services, including conventional and/or complementary forms of medicine.	National Association of Naturopaths of Chile, A.G	387	1. Environmental Health and Healthy Environments<br>2. Lifestyle<br>7. Management, quality and innovation	3. Good Health and Well-Being<br><br>10. Reduced Inequalities
Single, diverse and universal health system with recognition of non-conventional medicine and plurinationality.	To create a single health system which is diverse, universal, plurinational and integrated, to replace the current fragmented system.		118	1. Environmental Health and Healthy Environments<br>2. Lifestyle<br>7. Management, quality and innovation	3. Good Health and Well-Being<br><br>10. Reduced Inequalities
Occupational medicine (3)					
Including health and safety at work (SST) as a fundamental right for workers	Safety and security at work must be included as a fundamental right in the new constitution	Chilean Ergonomic Society, SOCHERGO	1353	1. Environmental Health and Healthy Environments<br>2. Lifestyle<br>7. Management, quality and innovation	3. Good Health and Well-Being<br><br>8. Decent Work and Economic Growth<br><br>10. Reduced Inequalities

Safety and health at work is a fundamental right according to OIT (International Labour Organisation). It is necessary to include it in the constitution	To establish the right of working in a healthy and safe space with minimum standards, with penalization to employers who do not abide.		244	1. Environmental Health and Healthy Environments<br>2. Lifestyle<br>7. Management, quality and innovation	3. Good Health and Well-Being<br><br>8. Decent Work and Economic Growth<br><br>10. Reduced Inequalities
Right to safety and health at work	The state must guarantee the right to safety and health for all workers in the country. The administration and coverage of these benefits will be the responsibility of the state and of non profit entities.	National Association of Workers from the Occupation Security Institute (ANATISEL)	189	1. Environmental Health and Healthy Environments<br>2. Lifestyle<br>7. Management, quality and innovation	3. Good Health and Well-Being<br><br>8. Decent Work and Economic Growth<br><br>10. Reduced Inequalities
Rare diseases and catastrophic situations (3)					
Mandatory and guaranteed coverage of health insurance in cases and diagnosis of rare diseases	All health insurances must guarantee to cover the costs associated to managing a rare disease		2866	5. Development and Disability<br>7. Management, quality and innovation	3. Good Health and Well-Being<br><br>10. Reduced Inequalities
Right to health by way of treatment of high cost diseases	To establish the right to guaranteed access to high cost treatments in the context of catastrophic diseases.		413	5. Development and Disability<br>7. Management, quality and innovation	3. Good Health and Well-Being

					10. Reduced Inequalities
Elective and free health for the catastrophically ill	Access both to public or private health must be free for terminally or catastrophically ill patients		115	5. Development and Disability<br>7. Management, quality and innovation	3. Good Health and Well-Being<br><br>10. Reduced Inequalities
Promotion and prevention in health (5)					
Constitution and Health: Modernization of the State	The constitution must put the person (or patient) at the centre of public policies, with prevention being the main focus of health policies, establishing a "National Preventive Health System"		289	1. Environmental Health and Healthy Environments<br>2. Lifestyle<br>7. Management, quality and innovation	3. Good Health and Well-Being<br><br>10. Reduced Inequalities
Right to health access (Disease prevention or free check-ups)	The state must guarantee access to health services, both public and private, with a preventative focus.		270	1. Environmental Health and Healthy Environments<br>2. Lifestyle<br>7. Management, quality and innovation	3. Good Health and Well-Being<br><br>10. Reduced Inequalities
Better health, lifestyle and wellness through physical education, exercise and recreation	To guarantee the physical and motor development of the population during the school stage, increasing hours of physical education. To guarantee promotion of physical and recreational activities for families, encouraging the active use of free time and leisure. To guarantee an adequate environment for the practice of physical		187	1. Environmental Health and Healthy Environments<br>2. Lifestyle<br>5. Development and Disability<br>7. Management, quality and innovation	3. Good Health and Well-Being<br><br>10. Reduced Inequalities

	activity and exercise for the entire population in general.				
Preventative health as a fundamental right	Preventive health must be guaranteed as a fundamental right and constitutional principle, focusing on nutrition and eliminating sedentarism.		162	1. Environmental Health and Healthy Environments<br>2. Lifestyle	3. Good Health and Well-Being<br><br>10. Reduced Inequalities
Right to knowledge and formation in first aid and basic health concepts	To guarantee education on first aid skill and on prevention of high incidence diseases in Chile		130	1. Environmental Health and Healthy Environments<br>2. Lifestyle<br>3. Communicable Diseases<br>4.Non communicable chronic diseases and Violence<br>7. Management, quality and innovation	3. Good Health and Well-Being<br><br>10. Reduced Inequalities
Incentivated Health	To incentivize sports and physical activity in order to develop mental and physical health.		45	1. Environmental Health and Healthy Environments<br>2. Lifestyle	3. Good Health and Well-Being<br><br>10. Reduced Inequalities
Waiting times in health (2)					
Diminish waiting times in access to public health services	The state must be expedient in helping citizens access health attention and medication. In cases where there is no availability, they must allow access to other professionals or systems		404	7. Management, quality and innovation	3. Good Health and Well-Being<br><br>10. Reduced Inequalities
Guarantees for prolonged waiting times in health	The state must provide aid in cases where waiting for health attention is prolonged, avoiding people's physical and mental health to be jeopardised.		173	7. Management, quality and innovation	3. Good Health and Well-Being

					10. Reduced Inequalities
Citizen participation (4)					
Right to an integral and participative health: creating wellbeing from the communities	The new constitution must contemplate a right to integral, quality, pertinent and opportune health for all people, with no discrimination and the state must commit to guaranteeing the community's systematic participation in public health strategies	Chilean Association of Music Therapy A. G.	541	7. Management, quality and innovation	3. Good Health and Well-Being<br><br>10. Reduced Inequalities
For a right to health based on the community	Health must be a right, with emphasis of territorial participation, with action originating from the communities, through a process of sustained participation with a focus on human rights.	Chilean Society of Communitarian Mental Health	483	7. Management, quality and innovation	3. Good Health and Well-Being<br><br>10. Reduced Inequalities
Binding citizen participation in health	Every person must have the autonomous right to participate and be part of the decision making processes in every area concerning public health	ANCOSALUD	184	7. Management, quality and innovation	3. Good Health and Well-Being<br><br>10. Reduced Inequalities
Towards an open, binding and feminist health	The new constitution must reformulate the structure and organisation of a new, equal and just health system, which must have a transversal perspective on health and includes active citizen participation.	Udumbara Collective	87	7. Management, quality and innovation	3. Good Health and Well-Being<br><br>5. Gender Equality<br><br>10. Reduced Inequalities
Health coverage and financing (11)					
Your health, your payment, your right	To propose a model where the State guarantees the right to health through multiple solidarity based alternatives, with a single universal plan, which incorporates		3410	7. Management, quality and innovation	3. Good Health and Well-Being

	the participation of different actors and a new scheme of regularisation, ensuring real supervision of public and private sectors.				10. Reduced Inequalities<br><br>16. Peace, Justice and Strong Institutions
Health without patches: Free, transparent and dignified	To establish a single insurance system that can be administered by public or private entities with additional complimentary insurances. To have free choice to choose between these providers.	Health without patches: Free and transparent	1935	7. Management, quality and innovation	3. Good Health and Well-Being<br><br>10. Reduced Inequalities<br><br>16. Peace, Justice and Strong Institutions
Right to Health	To establish the right to public and free health, where there is a single health system and ISAPRES are a complimentary insurances		1842	7. Management, quality and innovation	3. Good Health and Well-Being<br><br>10. Reduced Inequalities<br><br>16. Peace, Justice and Strong Institutions
Strengthening the health system, so that all Chilean people have access to quality and timely health benefits in an equitable manner	To ensure universal health coverage so that there is guaranteed access to health care, with no discrimination, ensuring that these services do not expose users to financial difficulties		990	7. Management, quality and innovation	3. Good Health and Well-Being<br><br>10. Reduced Inequalities

					16. Peace, Justice and Strong Institutions
Public, integrated health system	The constitution must guarantee a Public Health System, which replaces FONASA and the ISAPRE as we know them today and guarantees and opportune and integral health attention.		382	7. Management, quality and innovation	3. Good Health and Well-Being<br><br>10. Reduced Inequalities<br><br>16. Peace, Justice and Strong Institutions
The right to choose your insurance, health and education in public and private systems	The state must guarantee the freedom to choose which health insurance to have.		358	7. Management, quality and innovation	3. Good Health and Well-Being<br><br>10. Reduced Inequalities
To eliminate the obligatory health percentage of people in ISAPRES, leaving FONASA as the sole receptor	The obligatory percentage of people's income that goes towards health must necessarily go towards FONASA, leaving ISAPRE as a self financed option.		220	7. Management, quality and innovation	3. Good Health and Well-Being<br><br>10. Reduced Inequalities<br><br>16. Peace, Justice and Strong Institutions
You FONASA, me FONASA, we are all FONASA	Compulsory contribution for all to a single health fund and voluntary for isapres and/or complementary health insurance		162	7. Management, quality and innovation	3. Good Health and Well-Being<br><br>10. Reduced Inequalities

					16. Peace, Justice and Strong Institutions
Pension system and health insurance	Every citizen will have the freedom to choose the most convenient option for them to save for their pension. Equally, every citizen will have the freedom to choose the most convenient health insurance option for them or their family.		128	7. Management, quality and innovation	3. Good Health and Well-Being<br><br>10. Reduced Inequalities
Right to health and the presence of profit	To prohibit profit in health, both public and private		96	7. Management, quality and innovation	3. Good Health and Well-Being<br><br>10. Reduced Inequalities<br><br>16. Peace, Justice and Strong Institutions
0.1 of taxes to social security and health	0.1% of the taxes collected by the State must go to a common fund that goes to health (improve and maintain medical centers, emergencies, etc.) and another to ensure minimum guaranteed pensions		25	6. Emergencies and disasters<br>7. Management, quality and innovation	3. Good Health and Well-Being<br><br>10. Reduced Inequalities<br><br>16. Peace, Justice and Strong Institutions
Right to health and Universal health system (13)					
Single, Universal, Plurinational and Integrated Health System for the New	To transition to a single, universal, plurinational and integrated health system that replaces the fragmented and	Cabildo "Health, a right"	19852	7. Management, quality and innovation	3. Good Health and Well-Being

Chile that we are building democratically	inequitable system, where health access is based on subsidiarity, economic capacity and the profit of providers.				10. Reduced Inequalities<br><br>16. Peace, Justice and Strong Institutions
Health for a Chile that includes everyone	Health as a universal human right<br>Establish a unique and universal health system, with a supportive, equitable, participatory character, with a gender, intercultural, decentralized approach and articulated from the municipal territory.<br>Financing by progressive and proportional taxes.<br>Focused on APS and organized with network logic	National Confederation of Municipal Health Workers (CONFUSAM)	17294	1. Environmental Health and Healthy Environments<br><br>7. Management, quality and innovation	3. Good Health and Well-Being<br><br>10. Reduced Inequalities<br><br>16. Peace, Justice and Strong Institutions
FENPRUSS and the right to health	Right to health with a focus on social determinants of health and based on the principles of equity, universality, solidarity, comprehensiveness, interculturality, quality, efficiency and gender focus. In addition, there should be a universal health system financed by a single fund	Confederación FENPRUSS	16460	7. Management, quality and innovation	3. Good Health and Well-Being<br><br>5. Gender Equality<br><br>10. Reduced Inequalities<br><br>16. Peace, Justice and Strong Institutions
Universal Health System	Es deber del estado garantizar el derecho, acceso y accesibilidad a una salud digna e igualitaria a toda persona. Y para ello existirá un sistema de salud universal, que		4598	7. Management, quality and innovation	3. Good Health and Well-Being<br><br>10. Reduced Inequalities

	deberá garantizar la ejecución de las acciones de salud.				16. Peace, Justice and Strong Institutions
For decent health as a fundamental right for Chile	Health is a fundamental Human and Social Right that must be guaranteed by the State, for all people and communities that inhabit the national territory, without exclusion or discrimination of any kind.	Constituent Council for Health, Valparaíso	842	7. Management, quality and innovation	3. Good Health and Well-Being<br><br>10. Reduced Inequalities<br><br>16. Peace, Justice and Strong Institutions
2. Right to health: "First world medicine for everyone"	Every Chilean must have access to the best possible medical care, through which the "State protects free and equal access to actions for the promotion, protection and recovery of health and rehabilitation of the individual"		751	7. Management, quality and innovation	3. Good Health and Well-Being<br><br>10. Reduced Inequalities<br><br>16. Peace, Justice and Strong Institutions
Right to health: autonomy, dignified death, universal health system, binding participation, dignified attention and right to care	To reformulate the right to health, considering guaranteed access to a Public Health System at all levels of care, without discrimination of any kind. The financing, maintenance and operation of the Health System, as well as the professional training and territorial distribution of health workers shall be the responsibility of the State.		572	7. Management, quality and innovation	3. Good Health and Well-Being<br><br>10. Reduced Inequalities<br><br>16. Peace, Justice and Strong Institutions

A national health system for Chile: territorial, public, of quality and free	To guarantee a national health system for all, with specialists in every corner of the country, with commitment from the universities to train professionals. There must be a significant allocation of the state budget to implement a National Health Service.		424	7. Management, quality and innovation	3. Good Health and Well-Being<br><br>10. Reduced Inequalities<br><br>16. Peace, Justice and Strong Institutions
Universal health system from the moment of birth for the duration of life for all citizens, independently of their socioeconomic situation	There should be a universal health system for citizens which can be composed by existing health providers but are governed by a single body, ensuring a citizen is always covered throughout his life, especially in the case of a catastrophic illness.		416	7. Management, quality and innovation	3. Good Health and Well-Being<br><br>10. Reduced Inequalities<br><br>16. Peace, Justice and Strong Institutions
Right to public and private health	The right to health for all Chileans, coexisting public and private health.		340	7. Management, quality and innovation	3. Good Health and Well-Being<br><br>10. Reduced Inequalities<br><br>16. Peace, Justice and Strong Institutions
Health is a right	There must be free and quality health		195	7. Management, quality and innovation	3. Good Health and Well-Being<br><br>10. Reduced Inequalities

Universal Health, latest generation hospitals, how and what way to achieve and implement it in Chile	The new constitution must state that every person in Chile has the right to be treated free of charge or at low cost, by a Universal Health System		192	7. Management, quality and innovation	3. Good Health and Well-Being<br><br>10. Reduced Inequalities<br><br>16. Peace, Justice and Strong Institutions
"El Bosque" borough demands a universal health system conceived as a human right for everyone	Health as a human right guaranteed by the State of Chile, contemplating integrality, universality and justice in access and care for all.	Network of community leaders of El Bosque for a new constitution	191	7. Management, quality and innovation	3. Good Health and Well-Being<br><br>10. Reduced Inequalities<br><br>16. Peace, Justice and Strong Institutions
